# Polyethyleneimine-coated MXene quantum dots improve cotton tolerance to *Verticillium dahliae* by maintaining ROS homeostasis

**DOI:** 10.1038/s41467-023-43192-4

**Published:** 2023-11-15

**Authors:** Ping Qiu, Jiayue Li, Lin Zhang, Kun Chen, Jianmin Shao, Baoxin Zheng, Hang Yuan, Jie Qi, Lin Yue, Qin Hu, Yuqing Ming, Shiming Liu, Lu Long, Jiangjiang Gu, Xianlong Zhang, Keith Lindsey, Wei Gao, Honghong Wu, Longfu Zhu

**Affiliations:** 1 National Key Laboratory of Crop Genetic Improvement, Huazhong Agricultural University, Wuhan, 430070 People’s Republic of China; 2 Hubei Hongshan Laboratory, Huazhong Agricultural University, Wuhan, 430070 People’s Republic of China; 3 MOA Key Laboratory of Crop Ecophysiology and Farming System in the Middle Reaches of the Yangtze River, The Center of Crop Nanobiotechnology, Huazhong Agricultural University, Wuhan, 430070 People’s Republic of China; 4 State Key Laboratory of Cotton Biology, Henan Key Laboratory of Plant Stress Biology, Henan University, Kaifeng, 475004 People’s Republic of China; 5 School of Science, Huazhong Agricultural University, Wuhan, 430070 People’s Republic of China; 6 Department of Biosciences, Durham University, Durham, DH1 3LE UK; 7 Shenzhen Institute of Nutrition and Health, Huazhong Agricultural University, Wuhan, 430070 People’s Republic of China; 8 Shenzhen Branch, Guangdong Laboratory for Lingnan Modern Agriculture, Genome Analysis Laboratory of the Ministry of Agriculture, Agricultural Genomics Institute at Shenzhen, Chinese Academy of Agricultural Sciences, Shenzhen, 518120 People’s Republic of China

**Keywords:** Nanoparticles, Biotic, Comparative genomics, Agricultural genetics

## Abstract

*Verticillium dahliae* is a soil-borne hemibiotrophic fungal pathogen that threatens cotton production worldwide. In this study, we assemble the genomes of two *V. dahliae* isolates: the more virulence and defoliating isolate V991 and nondefoliating isolate 1cd3-2. Transcriptome and comparative genomics analyses show that genes associated with pathogen virulence are mostly induced at the late stage of infection (Stage II), accompanied by a burst of reactive oxygen species (ROS), with upregulation of more genes involved in defense response in cotton. We identify the V991-specific virulence gene *SP3* that is highly expressed during the infection Stage II. *V. dahliae SP3* knock-out strain shows attenuated virulence and triggers less ROS production in cotton plants. To control the disease, we employ polyethyleneimine-coated MXene quantum dots (PEI-MQDs) that possess the ability to remove ROS. Cotton seedlings treated with PEI-MQDs are capable of maintaining ROS homeostasis with enhanced peroxidase, catalase, and glutathione peroxidase activities and exhibit improved tolerance to *V. dahliae*. These results suggest that *V. dahliae* trigger ROS production to promote infection and scavenging ROS is an effective way to manage this disease. This study reveals a virulence mechanism of *V. dahliae* and provides a means for *V. dahliae* resistance that benefits cotton production.

## Abstract (web summary)

Verticillum wilt is an important cotton disease caused by fungal pathogen *Verticillium dahiae*. Here, the authors assemble the genomes of defoliating and non-defoliating isolates of the pathogen, identify virulence gene *SP3*, and develop a disease control strategy using polyethyleneimine-coated MXene quantum dots.

## Introduction

*Verticillium dahliae* is a soil-borne hemibiotrophic fungal pathogen that can infect more than 400 plant species, including major crops such as olive, tomato, lettuce, and cotton, causing significant yield losses^1^. The strains of *V. dahliae* from cotton can be divided into defoliating pathotypes, such as strain T-1 (later renamed T9), and nondefoliating pathotypes, such as strain SS-4^2^. In general, defoliating strains cause rapid and severe symptoms, such as leaf necrosis or even plant death, while nondefoliating strains induce only mild wilting symptoms^3^. The prevalence of highly virulent defoliating strains poses a major threat to cotton and olive around the world^4^. Thus, understanding variation in fungal pathogenicity at the genome level is essential to control plant disease.

Genomic structure variation is an important driver of the evolution of virulence in pathogens^5^. Lineage-specific (LS) genomic regions of pathogens are considered to be enriched with plant-induced genes, including those encoding secreted proteins (SPs)^5, 6^. For example, the avirulence factor *vdR3e* of *V. dahliae* race 3 was identified by functional genomics and found in a LS region^6^. Nowadays, more and more SPs that modulate the host defense response have been identified in pathogens^7, 8^. In the early stage of invasion, *V. dahliae* secretes chitin deacetylase (VdPDA1), which helps in the evasion of host immune recognition by modifying chitin oligosaccharides^7^. Necrosis and defoliation of leaves are typical symptoms following infection by defoliating *V. dahliae* isolates. Ethylene plays a vital role in plant defenses. Recently, it was found that cotton plants inoculated with the defoliating *V. dahliae* isolate V991 accumulate ethylene^9^. Transient inhibition of ethylene synthesis by knockdown of *ACO* expression through virus-induced gene silencing or attenuation of the ethylene signaling pathway through ectopic expression of *AtCTR1* in cotton improves resistance to *V. dahliae*, with little defoliation^9^. Moreover, the specific effector *PevD1* from *V. dahliae* can promote ethylene biosynthesis by interacting with and stabilizing the NAC transcription factor *ORE1* and activating expression of *ACS6*^10^. These results indicate that *V. dahliae* activate the ethylene signaling pathway for virulence and ethylene signaling is a susceptible factor. However, the molecular interaction between cotton and *V. dahliae*, especially defoliating isolates, still needs to be explored.

Metabolic reprogramming occurs in the host during pathogen colonization. Plant phenylpropanoid metabolites whose metabolic flux is redirected from the synthesis of lignin and flavonoids are required to achieve regulatory functions in plant development and plant-environment interactions^11, 12^. Knock-down of *GhLac1* leads to a redirection in metabolic flux in the phenylpropanoid pathway, causing the accumulation of jasmonic acid (JA) and secondary metabolites that confer tolerance to *V. dahliae* and cotton bollworm^13^. Pathogen‐induced plant reactive oxygen species (ROS) burst represents a hallmark of plant defenses^14^. Upon infection with avirulent pathogens, the rapid accumulation of ROS leads to necrosis of local cells, thus inhibiting the growth of pathogens^15^. Knock-down of the cotton cytochrome P450 *CYP82D* gene *GhSSN* significantly activates the ROS burst, eventually leading to plant necrosis and death^16^. However, the role of ROS homeostasis during the cotton-*V. dahliae* interaction remains unclear.

In this work, we identify SPs encoding genes that are found specifically in the defoliating isolate V991 through comparative genome analysis. Two distinct stages (Stage I and Stage II) are identified during the cotton-*V. dahliae* interaction through transcriptome analysis. Secondary metabolites play important roles in the host response in Stage I (from inoculation to 9 days post inoculation (dpi)). More SPs, including *SP3* specifically found in V991, are highly expressed from 12 to 21 dpi. We observe excessive accumulation of ROS in Stage II of infection. Knocking out of *SP3* results in a decreased accumulation of ROS and attenuates the virulence of V991. We design and synthesize polyethyleneimine-coated MXene (Ti_3_C_2_) quantum dots (PEI-MQDs) to alleviate ROS over-accumulation in infected cotton plants. We show that PEI-MQDs improve cotton resistance to *V. dahliae*. Thus, our work reveals that ROS is a susceptible factor and is enhanced by the pathogen and provides a nanobiotechnology approach that promotes verticillium wilt control in cotton.

## Results

### The presence and absence of genomic traits play a role in the pathogenicity difference in *V. dahliae*

**Fig. 1:**
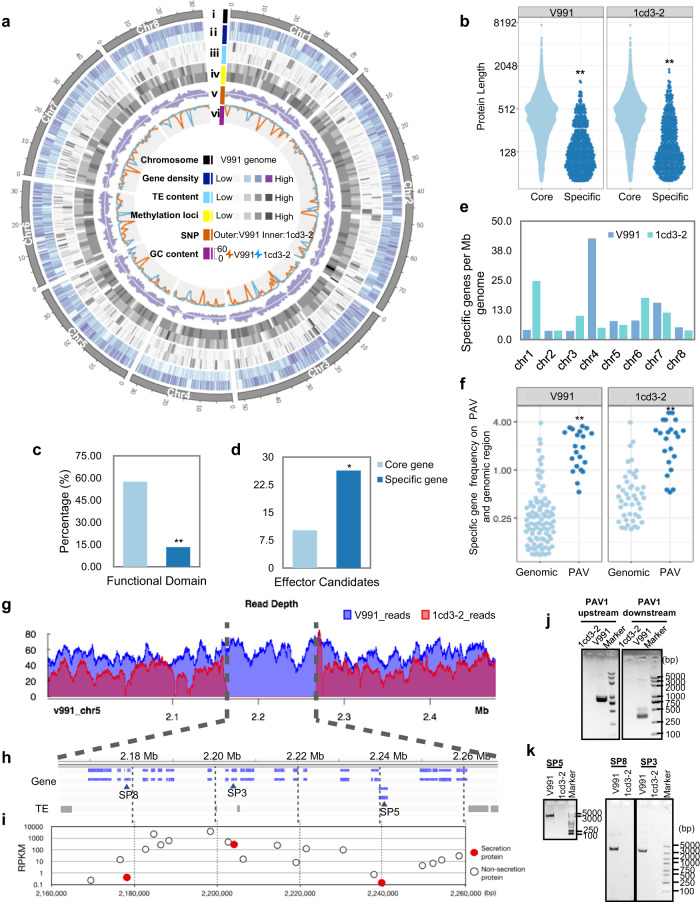
Comparative genomics analysis of two fungal isolates reveals PAVs. **a** Overview of the chromosomal features of V991 and 1cd3-2 with genetic and epigenetic data. i, Chromosomes of V991. ii, Gene density (bin=10 kb). Outer, V991; Inner, 1cd3-2. iii, TE content (bin=10 kb). iv, Methylation loci including 6 mA and 4mC (bin=100 kb). v, Distribution of SNPs (bin=100 kb). vi, GC content (bin=100 kb). Red line, V991; blue line, 1cd3-2. **b** Specific genes encoded smaller proteins with a significant *P* value < 2.2e-16 (Wilcox.test). **c** Specific genes were less likely to encode proteins with functional domains (own at least one Gene Ontology annotation). Statistical analyses were performed using Student’s *t* test: **P < 0.01. **d** Specific genes were more likely to encode candidate effectors (EffectorP classification). Statistical analyses were performed using Student’s *t* test: *P < 0.05. **e** Specific gene distribution in each chromosome. **f** In V991 and 1cd3-2, PAV regions had a higher frequency of specific genes than random genomic regions, with significant *P* values of 8.255e-12 and 8.839e-10, respectively (Wilcox test). **g–i** On chromosome 5, a large insertion incident (106 kb) affected 21 genes, including 20 specific genes, 3 of which encoded secreted proteins (SP3, SP5, SP8). **g** The PAV region was verified by PacBio sequence reads separately mapped to two genomes. **h** Gene and TE distribution in the PAV region. **i** Maximum gene regulation level for each gene from the upper panel. PCR validation of PAV regions (**j**) and specific genes (SP3, SP5, SP8) (**k**). **j**, **k** Experiments were repeated three times with similar results. Source data are provided as a Source Data file.

To understand the molecular basis of the virulence of *V. dahliae*, two *V. dahliae* isolates were selected: V991, a highly virulent and defoliating strain, and 1cd3-2, a nondefoliating strain causing mild symptoms (Supplementary Fig. 1). Their high-quality reference genomes comprising 8 chromosomes were assembled using single-molecule real-time sequencing (Fig. 1a). In total, 10,941 protein-coding genes for V991 and 10,971 protein-coding genes for 1cd3-2 were annotated (Supplementary Table 1). Furthermore, core gene and variant-specific gene sets were defined (Supplementary Table 2). Isolate-specific genes exhibited similar characteristics to newly evolved genes, with short protein lengths (Fig. 1b) and low transcriptional levels (Supplementary Fig. 2). The abundance of functional domains was significantly lower (Fig. 1c) while the ratio of candidate effectors was higher in the specific gene sets (Fig. 1d) compared to the core gene sets. In V991, most of the specific genes are located on chromosomes 4 and 7, and in 1cd3-2 on chromosomes 1 and 6 (Fig. 1e). Comparative genomics of presence and absence variations (PAVs) revealed that specific genes are more significantly associated with PAV regions than with random genomic regions (Fig. 1f and Supplementary Fig. 3). PAV regions comprise a small fraction of the fungal genome (5.2% ~ 8.1%), they carry more than one-third of specific genes (33.9% ~ 41.7%) (Supplementary Fig. 4). And specific genes represent a large fraction of genes in PAV regions (ranging from 69.1% to 72.9%) (Supplementary Fig. 4 and Supplementary Table 3). Overall, we detected 227 specific genes in PAV regions including 15 predicted SPs in V991 (Supplementary Table 4) and 193 specific genes including 16 predicted SPs in 1cd3-2. For example, 20 specific genes including 3 SPs (SP3, SP5 and SP8) were found in a large insertion of 106 kb on chromosome 5 in V991 (Fig. 1g–k).

### Transcriptome analysis identified two distinct stages in the cotton-*V. dahliae* interaction

Comparative genomics revealed that PAV regions are enriched with predicted effectors and SPs. To determine their expression during the interaction between *V. dahliae* and cotton, we performed a co-transcriptome analysis of cotton and *V. dahliae* with infected cotton hypocotyls over time (3, 6, 9, 12, 15, 18 and 21 dpi) (Supplementary Fig. 5). The cotton leaves showed no symptoms at 0-6 dpi but became chlorotic, necrotic and defoliated at 12-18 dpi upon inoculation with V991 (Supplementary Fig. 1a). Cotton infected with 1cd3-2 showed significantly milder Verticillium wilt symptoms (Supplementary Fig. 1a). The results of disease index analysis at different time points were consistent with the plant disease phenotypes (Supplementary Fig. 1b). The numbers of fungal transcripts were significantly higher from 12 dpi with V991 compared to those with 1cd3-2, indicating that the fungal biomass of V991 in the host was higher than that of 1cd3-2 at the late stage of infection (Supplementary Fig. 6).

**Fig. 2:**
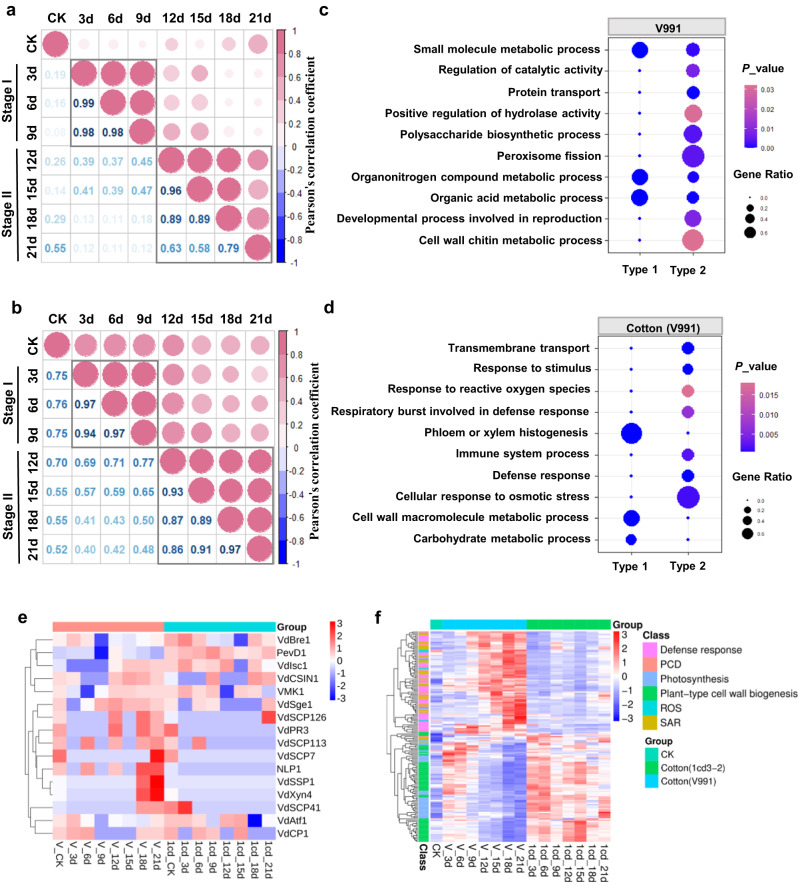
*In planta* transcriptome analysis of cotton-*V. dahliae* interactions. **a** Correlation analysis of the FPKM for differentially expressed genes (DEGs) in the V991 transcriptome between different time points from 3-21 days post infection (dpi). Pearson correlation of the FPKM was used to define the similarity of gene expression patterns among different time points. Values and colors indicated Pearson’s correlation coefficient. **b** Correlation analysis of the FPKM for DEGs in the cotton (V991) transcriptome between different time points from 3-21 dpi. **c** GO enrichment analysis of the stage-specific response to DEGs in the V991 transcriptome. DEGs that are upregulated at Stage I are named Type 1; DEGs that are upregulated at Stage II are named Type 2. The gene ratio is the number of DEGs divided by the total number of genes associated with a specific pathway. The color key corresponds to *p_*value for the enrichment score. *p_*value was determined by Fisher’s exact test. **d** GO enrichment analysis of stage-specific responses to DEGs in the cotton(V991) transcriptome. The color key corresponds to *p_*value for the enrichment score. *p_*value was determined by Fisher’s exact test. **e** Cluster heatmap of reported virulence-related genes in the V991 and 1cd3-2 transcriptomes. **f** Expression profiling of DEGs between two stages in cotton (V991) and cotton (1cd3-2) transcriptomes. CK indicates cotton seedlings without inoculation with V991 or 1cd3-2. Source data are provided as a Source Data file.

For the V991 transcriptome, correlation analysis of 2796 differentially expressed genes (DEGs) showed that the infection time points are separated into two stages: Stage I from 3 to 9 days (the early infection stage) and Stage II from 12 to 21 days (the late infection stage) (Fig. 2a). Similarly, correlation analysis of the 14940 DEGs of the cotton (V991) separated the infection time points into the same Stage I and Stage II (Fig. 2b). The fuzzy c-means clustering resulted in four distinct gene clusters (Supplementary Fig. 7). In the V991 transcriptome, Clusters 1-4 contained 646, 570, 677 and 903 DEGs, respectively. Clusters 1, 2, and 3 represent genes upregulated in Stage I (Type 1), and Cluster 4 represents genes upregulated in Stage II (Type 2) (Supplementary Fig. 7a). In the cotton (V991) transcriptome, Clusters 1-4 contained 4057, 3917, 4499 and 2467 DEGs, respectively. Cluster 1 represents genes upregulated in Stage I (Type 1), and Cluster 2 represents genes upregulated in Stage II (Type 2) (Supplementary Fig. 7b). The results of qRT-PCR analysis of selected genes with stage-specific expression patterns were consistent with the transcriptome results (Supplementary Fig. 8).

### The secreted protein SP3 is a key virulence factor in V991

Transcriptome analysis suggested that upregulated genes in V991 at Stage I were mainly involved in organonitrogen compound metabolism, organic acid metabolism and small molecule metabolism (Supplementary Data 1 and Fig. 2c). Upregulated genes in Stage II were mainly enriched in peroxisome fission, polysaccharide biosynthesis, regulation of catalytic activity, and developmental processes (Supplementary Data 2 and Fig. 2c). Moreover, more virulence-related genes, such as six types of genes predicted to encode effector, P450 (cytochrome P450), TCDB (membrane transport protein), Uniq gene, CAZY (carbohydrate active enzymes) and PHI (pathogen host interactions), were highly expressed at Stage II in V991 compared with 1cd3-2 (Supplementary Fig. 9). The predicted *SP*s in PAVs of V991 were also mainly expressed at Stage II (Supplementary Fig. 10). These results suggested that the highly expressed DEGs in V991 at Stage II, especially the virulence-related genes and *SP*s in PAVs, may determine virulence.

**Fig. 3:**
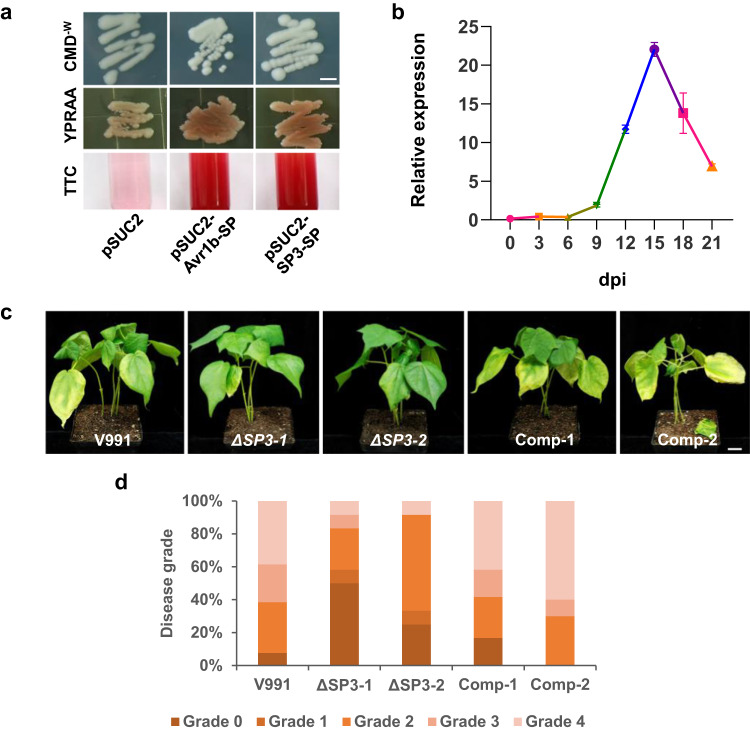
SP3 is a key virulence factor. **a** The signal peptide of SP3 is functional. Both YPRAA and TTC indicate successful secretion of invertase. The empty pSUC2 and pSUC2-Avr1b-SP vectors were used as negative and positive controls, respectively. The scale bar represents 5 mm. **b** Expression profile of SP3 during *V. dahliae* infection of cotton. Transcript levels of SP3 were determined by qRT–PCR. The experiment was repeated at least two times with similar results. Values are the means ± s.d. for three technical replicates. Transcript levels of genes were normalized to *v991_EVM0005718*. **c** Infection assays of wild-type V991, *ΔSP3* mutants, and *ΔSP3*/*SP3*-complementation transformants on cotton. The scale bar represents 2 cm. Three-week-old cotton seedlings were inoculated with 10^6 ^ml^−1^ conidia of the V991, *ΔSP3-1* and *ΔSP3-2* strains and complementary transformants Comp-1 and Comp-2. Photographs were taken at 18 dpi. **d** Disease index of infected cotton plants. Experiments were repeated three times with similar results. *n* = 15, *n* shows the number of samples. Source data are provided as a Source Data file.

The secreted protein SP3 in PAVs of V991 is a 372-amino acid protein with a secretion signal peptide and predicted as a quercetinase (Supplementary Table 4). The signal peptide of SP3 was demonstrated to be functional with the N-terminus of *Phytophthora sojae Avr1b* (pSUC2-Avr1b-SP) as a positive control (Fig. 3a). *SP3* was highly expressed *in planta* from 9 to 21 dpi, with a peak at 15 dpi (Fig. 3b). To determine SP3 function in fungal virulence, we generated two independent knock-out mutants (*ΔSP3-1*, *ΔSP3-2*) and two *ΔSP3*/*SP3*-complementation strains (Comp-1, Comp-2) of V991 (Supplementary Fig. 11). The *ΔSP3* mutants and complementation transformants showed normal growth and development as wild-type (Supplementary Fig. 11b). However, the *ΔSP3* mutants showed significantly reduced virulence, with fewer chlorotic leaves and a reduced disease index compared to the wild-type V991 and the complementation strains (Fig. 3c, d).

### The inability to maintain ROS homeostasis may be responsible for leaf chlorosis and necrosis

**Fig. 4:**
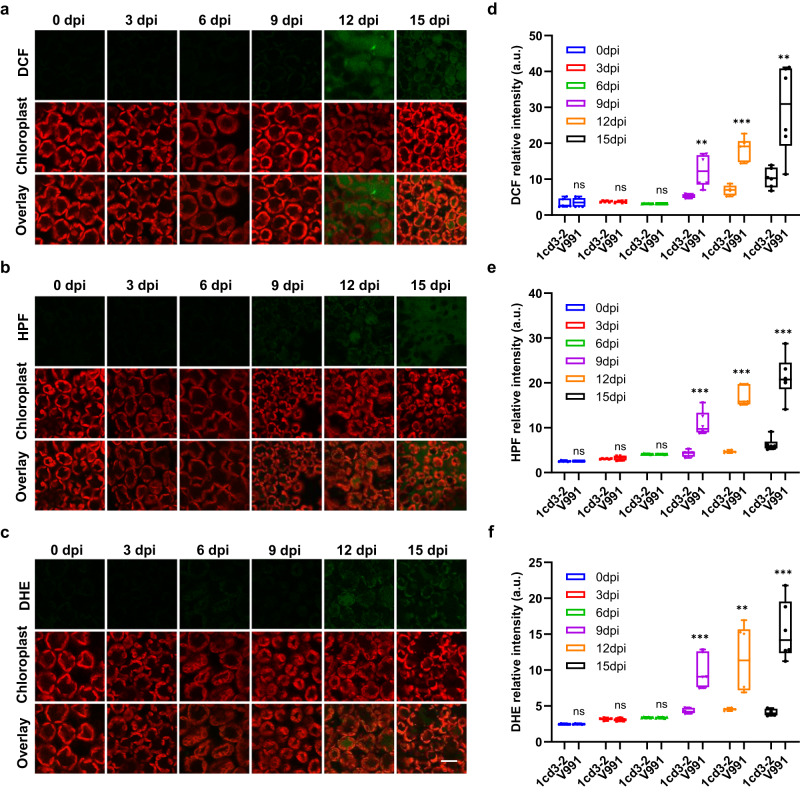
Dynamics of ROS in cotton infested with *V. dahliae* at different time points. **a** Confocal imaging of hydrogen peroxide (H_2_O_2_) (DCF) in cotton leaves infested with V991 at different time points. **b** Confocal imaging of hydroxyl radicals (OH·) (HPF) in cotton leaves infested with V991 at different time points. **c** Confocal imaging of superoxide anion (^•^O_2_^-^) (DHE) in cotton leaves infested with V991 at different time points, scale bar 50 μm. **d** Quantitative analysis of the fluorescence intensity of leaf hydrogen peroxide (H_2_O_2_) (DCF) in cotton infested with V991 and 1cd3-2 at different time points. **e** Quantification of the fluorescence intensity of hydroxyl radicals (OH·) (HPF) in cotton leaves infested with V991 and 1cd3-2 at different time points. **f** Quantification of the fluorescence intensity of superoxide anion (^•^O_2_^-^) (DHE) in cotton leaves infested with V991 and 1cd3-2 at different time points. In (**d**–**f**) values are the means ± s.d. for six biological replicates. *n* = 6, *n* shows the number of leaf samples. The asterisks indicate statistically significant differences between the two groups (ns, not significant; **P < 0.01; ***P < 0.001, Student’s *t* test). In each box and whisker plot, the centerline is the median. The bottom and top edges of the boxes indicate the twenty-fifth and seventy-fifth percentiles. Each dot represents individual data points. Source data are provided as a Source Data file.

DEGs at Stage I in cotton after inoculation with V991 were mainly enriched in cell wall metabolism and phloem or xylem histogenesis function (Supplementary Data 3 and Fig. 2d). DEGs at Stage II in cotton after V991 inoculation were mainly enriched in defense responses, stress responses and response to ROS (Supplementary Data 4 and Fig. 2d). Gene expression analysis also showed that more defense-related genes, such as various pathogenesis-related proteins (PRs), programmed cell death (PCD) and ROS-related genes, were induced at Stage II in cotton after inoculation with V991 compared with 1cd3-2 (Fig. 2f). The fluorescent dyes DCF (2′,7′-dichlorodihydrofluorescein diacetate, indicating H_2_O_2_), DHE (dihydroethidium, indicating ^•^O_2_^-^) and HPF (2-[6-(4′-Hydroxy) phenoxy-3H-xanthen-3-on-9-yl] benzoic acid, indicating OH^•^) were employed for confocal imaging to evaluate ROS homeostasis during the cotton-*V. dahliae* interaction (Fig. 4). We observed no or faint green fluorescence in cotton leaves at Stage I, indicating low levels of ROS (Fig. 4). ROS accumulation started at 9 dpi and increased significantly to high levels at Stage II infected with V991 (Fig. 4), concomitant with proliferation of V991 *in planta* (Supplementary Fig. 6) and disease symptoms of chlorosis, necrosis, and defoliation (Supplementary Fig. 1). While the level of ROS in plants infected with 1cd3-2 was significantly lower than that with V991 (Fig. 4d–f). These results suggested that V911 cause high ROS accumulation in cotton, which result in severe disease symptoms.

### PEI-MQDs decrease ROS to improve cotton tolerance to *V. dahliae*

To scavenge ROS, we designed and synthesized polyethyleneimine-coated MXene (Ti_3_C_2_) quantum dots (PEI-MQDs). Supplementary Figure 12a shows that PEI-MQDs are well dispersed and that the core of the synthesized PEI-MQDs is spherical. The inset image shows that the d-spacing of the synthesized PEI-MQDs is 0.229 nm (Supplementary Fig. 12a). The zeta potential of the PEI-MQDs was calculated to be 31.71 ± 2.14 mV, with an average diameter of 3.85 ± 1.5 nm (Supplementary Fig. 12b). PEI-MQDs had the maximum emission peak at 475 nm under 340 nm excitation (Supplementary Fig. 12c). The scavenging efficiencies of 50 mg/L PEI-MQDs for ^•^O_2_^-^, H_2_O_2_, OH^•^ and ONOO^−^ were 11.70%, 6.56%, 42.93% and 27.23%, respectively, in vitro (Supplementary Table 5). As shown in Supplementary Figure 12d, 50 mg/L PEI-MQDs were exposed to H_2_O_2_ solution for 24 hours, and then the isolated PEI-MQDs were tested in vitro for H_2_O_2_-scavenging activity. The results showed that the PEI-MQDs retained the ability to scavenge H_2_O_2,_ indicating that PEI-MQDs may act as nanozymes (Supplementary Fig. 12d). Further colorimetric reaction confirmed that the PEI-MQDs exhibited peroxidase-like activity (Supplementary Fig. 12e-f). In addition, there was no significant effect on the radial growth of V991 on PDA (potato dextrose agar) agar plates four days after exposure to 50 mg/L PEI-MQDs compared with the control (Supplementary Fig. 12g-h), which demonstrates that the PEI-MQDs do not inhibit the pathogen development. To verify that PEI-MQDs can enter cotton cells, the PEI-MQDs were labeled with fluorescent fluorescein isothiocyanate (3’, 6’-dihydroxy-5-isothiocyanato-3H-spiro[isobenzofuran-1,9’-xanthen]−3-one, FITC) and infiltrated into roots. After 3 h of incubation, infiltration with FITC-PEI-MQDs was assessed by confocal imaging. No fluorescence signals were detected in the control, and the colocalization rate between FITC-PEI-MQDs and the membrane was 72.21 ± 13% (Supplementary Fig. 12i-j).

**Fig. 5:**
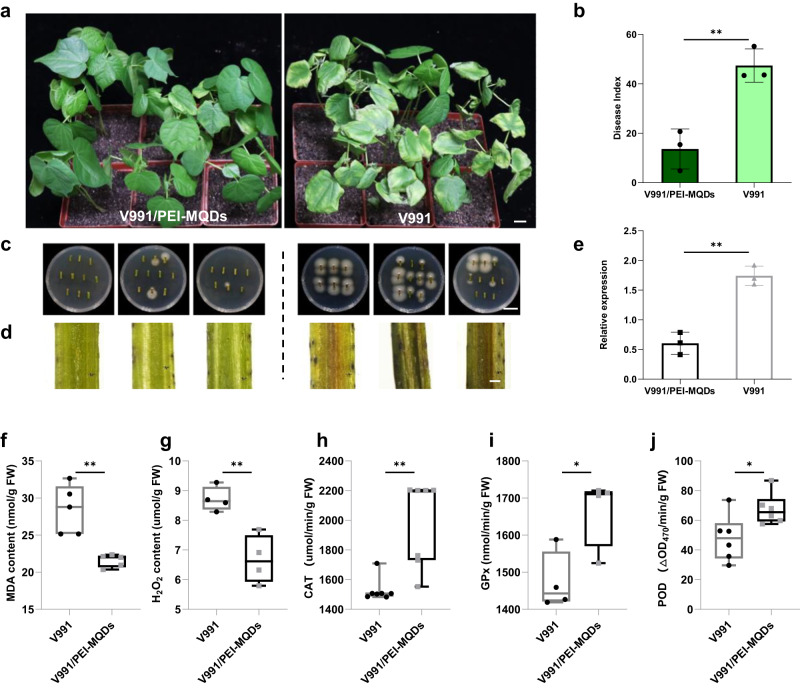
PEI-MQDs improve cotton tolerance to *V. dahliae* by maintaining ROS homeostasis. **a** Disease symptoms of cotton plants at 18 days after V991 inoculation with and without PEI-MQDs treatment. The scale bar represents 2 cm. **b** Disease index of infected cotton plants. **c** Fungal recovery assay. The scale bar represents 2 cm. **d** Sections from the cotyledonary node of cotton at 18 d after *V. dahliae* infection. The scale bar represents 100 μm. Experiments were repeated three times with similar results. **e** qPCR analysis of the amount of fungal DNA. *GhUB7* was used as an internal reference, and the *ITS* of the fungal ribosomal DNA was targeted. In (**b**) and (**e**), values are the means ± s.d. for three biological replicates. Asterisks indicate statistically significant differences between the two groups (**P < 0.01, Student’s *t* test). Each dot represents individual data points. **f**–**j** PEI-MQDs reduced MDA and H_2_O_2_ content and increased catalase (CAT), glutathione peroxidase (GSH-Px) and peroxidase (POD) activities in the PEI-MQDs treated plants at 12 dpi. **f** MDA content. **g** H_2_O_2_ content. **h** CAT activity. **i** GPx activity. **j** POD activity. **f**–**j** Values are the means ± s.d. at least three biological replicates. *n* = 3, *n* shows independent experiments. Asterisks indicate statistically significant differences between the two groups (*P < 0.05, **P < 0.01, Student’s *t* test). In each box and whisker plot, the centerline is the median. The bottom and top edges of the boxes indicate the twenty-fifth and seventy-fifth percentiles. Each dot represents individual data points. Source data are provided as a Source Data file.

Notably, cotton pretreated with PEI-MQDs showed fewer disease symptoms than the control (Fig. 5a). Consistent with this, the corresponding disease index, fungal recovery assay, observed browning of vascular tissue, and measurement of fungal biomass (Fig. 5b–e) all supported that PEI-MQDs increased cotton tolerance to *V. dahliae*. The MDA (malondialdehyde) and H_2_O_2_ contents in PEI-MQDs-treated cotton leaves were also significantly lower than those in the control (Fig. 5f, g). In addition, we observed higher activities of catalase (CAT), glutathione peroxidase (GSH-PX), and peroxidase (POD) in the PEI-MQD-treated plants compared with the control plants at 12 dpi (Fig. 5h-j), indicating that the PEI-MQDs enhanced the activities of these enzymes. Furthermore, levels of ROS were measured in cotton treated with PEI-MQDs at different timepoints (Supplementary Fig. 13). The fluctuation of ROS in plants caused by V991 and PEI-MQDs was not significant (Supplementary Fig. 13a, b), and the overall ROS level in plants was still very low and thus not induced at the early stage (6 dpi) (Supplementary Fig. 13b, d, h). However, at the late stage (12 dpi), excessive ROS were detected in seedlings inoculated with V991 (Supplementary Fig. 13c). When treated with the PEI-MQDs, levels of ROS were significantly reduced at the late stage (Supplementary Fig. 13c, d, h). Thus, ROS-scavenging PEI-MQDs can improve cotton tolerance to *V. dahliae* by maintaining ROS homeostasis at the late stage of infection.

## Discussion

Due to its asexual lifestyle, large-scale genomic rearrangements, horizontal gene transfer, and transposable element (TE) activity mainly contribute to the genomic diversity of *V. dahliae*^17, 18^. Genomic structure variation (SV), such as presence/absence variants (PAVs), is an important driver of genome evolution to confer virulence in pathogens^5^. In our study, comparative genomics between defoliating isolate V991 and nondefoliating isolate 1cd3-2 showed that PAVs are associated with virulence-related genes. For example, on chromosome 5, the most induced gene was v991_EVM0005595 (Nmr2) (Fig. 1i), part of the NADPH-dependent genetic switch regulating plant infection. NADP-dependent Nmr transcriptional corepressors derepress expression of a set of virulence-associated genes during appressorium-mediated initiation of rice blast disease^19^. On chromosome 7, there are four specific secretion proteins, which were all upregulated (Supplementary Fig. 10 and Supplementary Fig. 14). These observations suggest that PAV variants be associated with specific genes, which contain distinctive variations and further lead to functional divergence between fungal strains.

Regarding the interaction between *V. dahliae* and cotton, previous studies have mostly focused on either the pathogen or the host at the early infection stage^20^. In the present study, we found two distinct stages during the molecular interaction through time-course transcriptome analysis. *VdCP1*^21^, which suppresses host defense responses, was specifically induced during Stage I, and some genes related to metabolism such as *VdAtf1*^22^ and *VdBre1*^23^, were also induced during Stage I (Fig. 2e), which mainly provide nutrition to the colonization. However, many genes related to microsclerotia (*VdPR3*^24^) and mycelial growth (*VdSge1*^25^, *VMK1*^26^, *VdCSIN1*^27^) were induced during Stage II (Fig. 2e). A large number of effectors, such as *VdSCP7*^28^, *VdNLP1*^29^ and *VdXyn4*^30^, which accelerate plant death were highly expressed during Stage II (Fig. 2e). These results suggest that *V. dahliae* transitions from a biotrophic to a necrotrophic phase during the host-pathogen interaction. Nevertheless, how these two stages are transformed remains to be investigated.

**Fig. 6:**
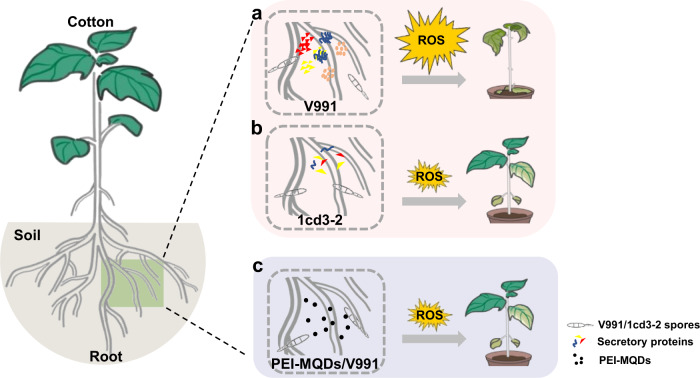
The interaction model between *V.dahliae* and cotton. **a** V991 regulates expression of more virulence-related genes during the host interaction, causing excessive accumulation of ROS in cotton and leading to severe disease. **b** 1cd3-2 expressed fewer virulence-related genes during the host interaction, resulting in lower accumulation of ROS in cotton and ultimately a milder phenotype of disease. **c** PEI-MQDs increase resistance to V991 by maintaining ROS homeostasis in cottons.

The specific secretory protein SP3 was identified as a key virulence factor and was highly expressed in the late stage of infection. Protein function prediction indicates that SP3 is a quercetinase. It has been reported that a quercetinase, VdQase, is involved in the catabolism of quercetin and other flavonols *in planta*, with a higher accumulation of flavonols in the stems and a higher concentration of rutin in the leaves of potato infected by *VdQase* mutants^31^. As antioxidants, flavonoids such as rutin play an important role in scavenging ROS^32^. With the *SP3* knockout mutants, levels of ROS at Stage II in cotton leaves infected were significantly lower than those in cotton leaves infected with V991 (Supplementary Fig. 15). Therefore, we speculate that a high expression level of *SP3* in Stage II promotes accumulation of more ROS and thus accelerates host death. In addition to *SP3*, other specific SPs and effectors were also highly expressed during Stage II (Supplementary Fig. 9e and Fig. 2e), such as *VdXyn4*^30^ and *VdNLP1*^29^. As described in the interaction model (Fig. 6a, b), more effector proteins were secreted by V991, which caused ROS accumulation in cotton, leading to susceptibility; conversely, 1cd3-2 secreted fewer effector proteins during invasion, and cotton showed a relatively mild phenotype. How these effectors are co-expressed during phase transition needs to be explored further.

Previous interaction analysis between plants and *V. dahliae* found that cell wall structure and lignin metabolism play an important role in disease resistance, with several genes involved in cell wall or lignin synthesis being identified. For example, overexpression of *GhLac1* leads to increased lignification in cotton, which enhances tolerance to *V. dahliae*, bollworms and aphids^13^. In this study, genes associated with cell wall synthesis were significantly activated during Stage I after inoculation with highly pathogenic pathogens. This is consistent with most research reports. We also found that after inoculation with less virulent pathogens, cotton showed significant cell wall synthesis throughout the entire interaction process (Fig. 2f). This proves that cell wall lignification confers resistance to pathogens during the interaction. Intracellular ROS homeostasis is critical for plant defense. In mammals, excessive activation of the immune response leads to inflammatory diseases^33^. Similarly, in plants, excessive accumulation of ROS can affect plant growth and development or even threaten survival^16, 34^. A recent study reported that the cell death triggered by ROS is the main reason for Huanglongbing (HLB) symptom^32^. At Stage II following inoculation with V991, the transcripts of a large group of defense-related genes, such as pathogenesis-related genes (*PR*s), were upregulated in the host, along with PCD induction and ROS accumulation (Fig. 2f). Infected cotton leaves showed significantly higher levels of ROS compared with Stage I (Fig. 4). The H_2_O_2_ content and MDA content in V991-infected cotton leaves were significantly increased compared to healthy plants at 12 dpi (Supplementary Fig. 16a, b). These results suggest that the disease symptoms of leaf chlorosis and necrosis may be related to excessive ROS accumulation in cotton induced by defoliating strain of *V. dahliae*.

Studies have shown that some nanomaterials can simulate the activity of antioxidant enzymes, with an increased ability to scavenge ROS, promotion of the germination and growth of plants, and enhanced plant resistance^35, 36^. For example, CeO_2_ nanoparticles modulate Cu–Zn superoxide dismutase and lipoxygenase-IV isozyme activities to alleviate membrane oxidative damage to improve rapeseed salt tolerance^35^. Zinc oxide nanospheres significantly reduce the severity of bacterial wilt and increase plant biomass and antioxidant enzyme content in tomato^36^. Our results show that ROS-scavenging PEI-MQDs enabled the maintenance of ROS homeostasis in infected cotton plants and protected cotton plants from *V. dahliae* (Fig. 6c). Our work provides insights into the molecular interaction between cotton and *V. dahliae* and proposes a nanobiotechnology approach to protect cotton plants from *V. dahliae*.

## Methods

### Genome sequencing and assembly

Commercial DNA extraction kit (Tiangen Biotech, Beijing, China) was used to extract genomic DNA from *Verticillium dahliae* isolates V991 and 1cd3-2 for long read sequencing. Twenty kilobase-long-insert libraries were sequenced with PacBio RS II SMRT cells. Canu was used to assemble long reads for genome assembly^37^. Illumina sequencing of 500-bp-insert libraries was used to refine the initial assembly genomes.

### TE annotation

Species-specific libraries were used for annotating repeats for each isolate. LTR_FINDER (version 1.05), MITE-Hunter (20100819), Repeat Scout (version 1.0.5) and PILER-DF (version 2.4) were utilized to generate repeat libraries based on a structure-based method and de novo prediction^38^. PASTE Classifier (version 1.0) was applied for repeat classification and then merged with all repeats from Repbase Database (version 19.06)^39^. Finally, Repeat Masker was used to predict fungal isolate repeats^40^.

### Gene prediction and annotation

EVM was used to integrate all predicted results^41^. For de novo prediction, several software programs were used, including Augustus, GlimmerHMM, and SNAP (version 2006-07-28), to scan the repeat-masked genome^42^. For the homolog-based approach, GeMoMa was used based on *Verticillium dahliae* VdLs.17, *Verticillium alfalfae* VaMs.102, *Fusarium oxysporum* f. sp. lycopersici 4287, *Fusarium verticillioides* 7600, and *Fusarium graminearum* PH-1^43^. PASA was used to predict genes based on Illumina sequencing RNA data^44^.

### DNA methylation modification identification

Based on PacBio-generated raw data, 6 mA and 4mC base modifications were identified using PacBio SMRT Analysis 2.3.0 with default settings (https://www.pacb.com/documentation/smrt-analysis-software-installation-v2-3-0/).

### Isolate specific genes identification

Based on bidirectional BLAST results, homologous gene pairs (smaller than E-value 1e-20) were retained as core gene sets. Extra gene sets were defined as specific genes to represent the functional specificity of one genome. PCR primers were separately designed based on the gene upstream 1 Kb and downstream 1 Kb to cover gene regions (Supplementary Data 5).

### Present/absent variation (PAV) identification

The program Nucmer in Mummer was used to compare two genomes, and then “show-diff” was utilized to identify PAVs. Candidate PAVs were filtered by abandoning sequences homologous with the other genome (threshold: E < 1e-5, coverage>50% and identity>90%). To ensure accuracy, candidate PAVs were aligned with the Pacbio-sequenced raw reads of the other genome and deleted candidates with similar thresholds (E < 1e-5, coverage>60% and identity>90%). Finally, unaligned regions were regarded as lineage-specific regions.

### Pathogen cultivation, infection and disease assay

*V. dahliae* was inoculated on a potato dextrose agar (PDA) plate and placed in an incubator at 25 °C for four days. Fungal colonies were transferred into Czapek medium on a shaker at 120 rpm at 25 °C for four days until the spore concentration reached ~10^8^ spores mL^−1^. Cotton (*Gossypium hirsutum cv*. Jin668) was watered with Hoagland solution for 3 weeks under greenhouse conditions of 25 °C to 28 °C under long-days with an 8 h/16 h dark/light photoperiod and a relative humidity of 60%. Three-week-old cotton seedlings were inoculated for two minutes with 1 × 10^6^ conidia mL^−1^ by the root-dip method using a spore suspension, and the inoculated plants were placed in pots (10 × 10 cm) filled with standard soil mix (Xingyuxing, Wuhan, China). The disease index was scored using at least 25 plants per treatment and repeated at least three times^45^. The disease grade was classified as follows: 0 (no symptoms), 1 (0%–25% wilted leaves), 2 (25%–50% wilted leaves), 3 (50%–75% wilted leaves) and 4 (75%–100% wilted leaves).

### Generation of gene deletion mutants and mutant complementation

Performing *A. tumefaciens*-mediated *V. dahliae* transformation and PCR-based transformants screening to generate the knockout mutants^28^. To produce complementary transformants, the coding sequence for *SP3* with its native promoter was cloned and inserted into the binary vector p823-GFP encoding resistance to G418, and *SP3* was reintroduced into the *ΔSP3* strains. Complemented transformants were obtained using an *A. tumefaciens* -mediated transformation method^28^. All primers used are shown in Supplementary Data 5.

### Yeast signal sequence trap system

The predicted signal peptide of SP3 was cloned and inserted into pSUC2T7M13ORI (pSUC2), and the resulting plasmid was transformed into Yeast YTK12 strains. All strains were screened on CMD-W medium (0.67% yeast N base without amino acids, Trp dropout supplement, 2% sucrose, 0.1% glucose, and 2% agar), and positive transformants were incubated on YPRAA medium (1% yeast extract, 2% peptone, 2% raffinose, 2% agar, pH 5.8)^46^. Invertase enzymatic activity was assessed by a 2,3,5-triphenyltetrazolium chloride (TTC) reduction assay^47^. Total yeast cells were collected and washed with double distilled water. The pellet was resuspended in 0.1% colorless TTC dye and incubated at 30 °C for 1-2 h, and the color change was determined at room temperature. The empty pSUC2 and pSUC2-*Avr1b*SP vectors were used as negative and positive controls, respectively.

### Total RNA extraction and RT‒qPCR analysis

Total RNA was extracted using an RNA Extraction Kit (Tiangen Biotech, Beijing, China). First-strand cDNA was generated from 3 μg of total RNA using SuperScript III reverse transcriptase (Invitrogen, Carlsbad, CA, USA) and diluted 100 times with double distilled water. Reverse transcription quantitative PCR was performed using a 7500 Real Time PCR system (ABI, Foster City, CA, USA) in a 15 μL reaction volume. qRT**‒**PCR was performed as follows: an initial 95 °C denaturation step for 3 min, followed by 40 cycles of 95 °C for 15 s and 60 °C for 45 s. The housekeeping genes *GhUB7* and *v991_EVM0005718* were used as internal controls for cotton and *V. dahliae*, respectively. The primers used in this study are listed in Supplementary Data 5.

### Sample preparation and RNA-seq

We conducted bidirectional transcriptome analysis on hypocotyls of cotton during the whole period of interaction (0, 3, 6, 9, 12, 15, 18, 21 dpi). RNA was isolated from hypocotyls of cotton infected with *V. dahliae*. The library preparations were sequenced using the Illumina NovaSeq 6000 platform, and 150-bp paired-end reads were generated. Sequencing adapters were removed, and consecutive low-quality bases were trimmed from both the 5’ and 3’ ends of the reads. High-quality RNA-Seq reads were aligned to the *V. dahliae* genome using HISAT2 with default parameters. Transcripts of each sample were assembled using Stringtie, and pairwise comparisons between samples were performed using the edgeR package^48^. We used FPKM > 0.01, fold change≥2 and *P* < 0.05 as thresholds to screen for DEGs. The RNA-Seq reads were also processed and mapped to cotton in the same way. We used FPKM > 1, fold change≥2 and *P* < 0.05 as the thresholds to screen for DEGs in the cotton transcriptomes. The threshold *P* < 0.05 was selected to identify significantly enriched GO terms.

### Synthesis and characterization of polyethyleneimine-coated MXene quantum dots

Polyethyleneimine-coated MXene quantum dots (PEI-MQDs) were synthesized from Ti_3_C_2_ MXene powder (Jilin Science and Technology) and 25 mg of polyethyleneimine (PEI) (Sigma, MV 1800). Briefly, the mixture of Ti_3_C_2_ MXene powder and PEI was added to 10 mL DI water and then perfused with N_2_ for 5 min The solution was then incubated at 120 °C for 8 h in a drying oven. The pH of the obtained solution was adjusted to approx. 7.0, and the final solution was dialyzed for 24 h. After dialysis, the solution was freeze-dried for 24 h, and the final products were stored in a refrigerator until further use. For nanoparticle characterization, transmission electron microscopy (TEM) images were captured by an H-7650 (HITACHI, Japan). The surface charge of PEI-MQDs was measured by using a zetasizer (NanoBrook 90Plus Zeta). The ζ-potential characterization (NanoBrook 90Plus Zeta) confirmed the presence of a negative charge for PEI-MQDs.

### Catalytic activity of PEI-MQDs

The catalytic activity of PEI-MQDs was evaluated by measuring their peroxidase-mimicking activities with the use of the colorimetric substrate 3,3,5,5-tetramethylbenzidine (TMB), which is oxidized in the presence of H_2_O_2_ to produce a color reaction^49^.

### Root absorption and FITC-PEI-MQDs application to cotton plants

Cotton roots were incubated in a solution containing PEI-MQDs (50 mg/L), with the root completely submerged in the dark for 3 h. The PEI-MQDs solution was removed, and the roots were placed in water for one day before inoculation with V991. Two treatment replicates were divided into two groups: each group had at least 16 plants, one group treated with FITC-PEI-MQDs and another with water as a control. The mixture was composed of 200 μL anhydrous ethanol, 50 FITC (2.5 mg/mL), 1 mL 50% alcohol and 2 mg/mL PEI-MQDs, and kept in a 20 mL glass vial at 1000 × g for 10 min in the dark. The resulting mixture was purified using a 10-kDa filter (4200 × g for 5 min at least 8 times) to remove the dissociated chemicals. The final solution was labelled with FITC-PEI-MQDs and stored at 4 °C until use.

### ROS determination and enzyme activity assay

DCF, DHE and HPF fluorescent dyes are used to visualize ROS in leaf tissues^50^. Leaf discs (diameter, 5 mm) were incubated with either 25 μM DCF for 30 min or 10 μM DHE dye for 30 min or 10 μM HPF for 1 h in darkness (in TES infiltration buffer, pH 7.5). After incubation, the leaf discs were rinsed with deionized water three times and mounted onto a glass slide. A coverslip was placed on the top to seal the well while ensuring that no air bubbles remained trapped. The samples were imaged using a Leica SP8 confocal microscope (Leica Microsystems, Germany). Six repetitions were performed. The fluorescence intensities of DHE, DCF and HPF were calculated by LAS (Leica Application Suite) AF Lite software.

Hydrogen peroxide (H_2_O_2_), malondialdehyde (MDA), catalase (CAT), glutathione peroxidase (GSH-PX) and peroxidase (POD) were analyzed using H_2_O_2_, MDA, CAT, GSH-PX and POD assay kits (Suzhou Grace Biotechnology Co., Ltd.).

### Reporting summary

Further information on research design is available in the Nature Portfolio Reporting Summary linked to this article.

### Supplementary information

Supplementary Information

Description of Additional Supplementary Files

Supplementary Data 1

Supplementary Data 2

Supplementary Data 3

Supplementary Data 4

Supplementary Data 5

Reporting Summary

### Source data

Source Data

## Data Availability

All data generated from this study are available in the article and Supplementary Information files. The raw sequences of V991 and 1cd3-2 have been deposited in the NCBI Bioproject database under accession PRJNA510201. The V991 and 1cd3-2 genome assembly data have been deposited in the Genome Warehouse in National Genomics Data Center, Beijing Institute of Genomics, Chinese Academy of Sciences/China National Center for Bioinformation under accession number PRJCA020793 and PRJCA020800, respectively. The raw RNA-seq data generated in this paper have been deposited in the Genome Sequence Archive in the National Genomics Data Center, China National Center for Bioinformation/Beijing Institute of Genomics, Chinese Academy of Sciences under accession CRA008562. Source data are provided with this paper.
