# Supplementary material for: Polyethyleneimine-coated MXene quantum dots improve cotton tolerance to Verticillium dahliae by maintaining ROS homeostasis
Source: Nat Commun. 2023 Nov 15;14:7392. doi: 10.1038/s41467-023-43192-4 (PMC10651998; doi:10.1038/s41467-023-43192-4)
Supplement: Supplementary file 1 — Supplementary Information [file 41467_2023_43192_MOESM1_ESM.pdf]

Polyethyleneimine coated MXene quantum dots improve cotton tolerance to *Verticillium dahliae* by maintaining ROS homeostasis

Qiu *et al.*

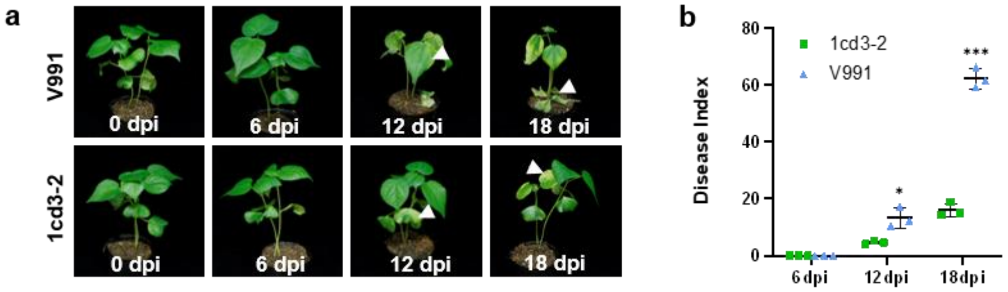

Supplementary Fig. 1. The pathogenicity comparison of two reference field isolates. a The phenotype of cotton seedlings when inoculated by V991, regarded as defoliating isolate and the phenotype of cotton seedlings when inoculated by 1cd3-2, regarded as non-defoliating isolate at different time points. **b** The disease index of cotton inoculated with V991 and 1cd3-2 at different time points. The values are the means ± s.d. for three biological replicates. n = 15, n shows the number of samples. The asterisks indicate statistically significant differences between the two groups (*P<0.05, ***P<0.001, Student's t-test). Each dot represents individual data points. Source data are provided as a Source Data file.

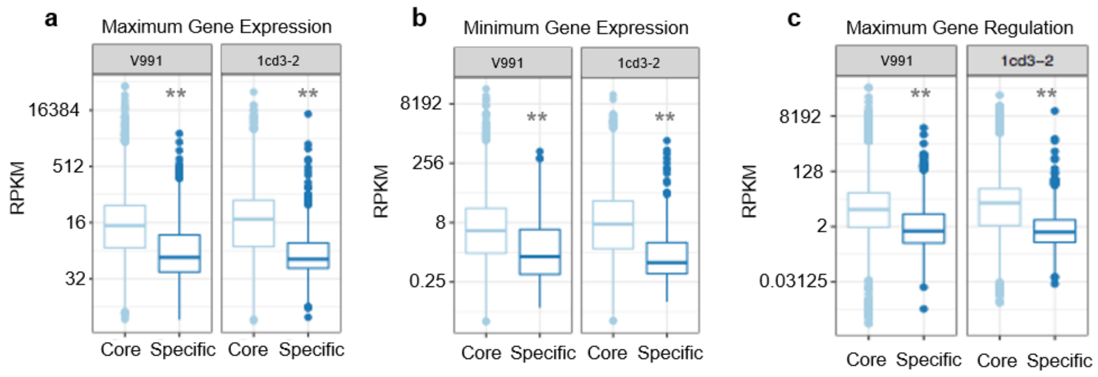

Supplementary Fig. 2. specific genes have lower transcriptional levels. a The transcripts of specific genes were more lower during the whole infection stage with significant *P*-value < 2.2e-16 (Wilcox.test). **b** Specific gene have higher Minimum transcription level with significant *P*-value < 2.2e-16 (Wilcox.test). **c** Specific gene's expressional changing level (the difference between Maximum and Minimum expression) is higher with significant *P*-value < 2.2e-16 (Wilcox.test). In each box and whisker plot, the center line is the median. The bottom and top edges of the boxes indicate the twenty-fifth and seventy-fifth percentiles. Source data are provided as a Source Data file.

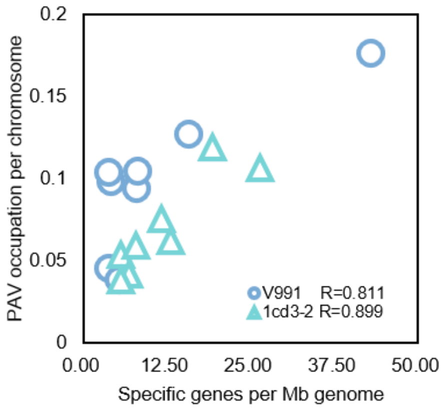

Supplementary Fig. 3. High correlation of specific gene location and PAV location. Source data are provided as a Source Data file.

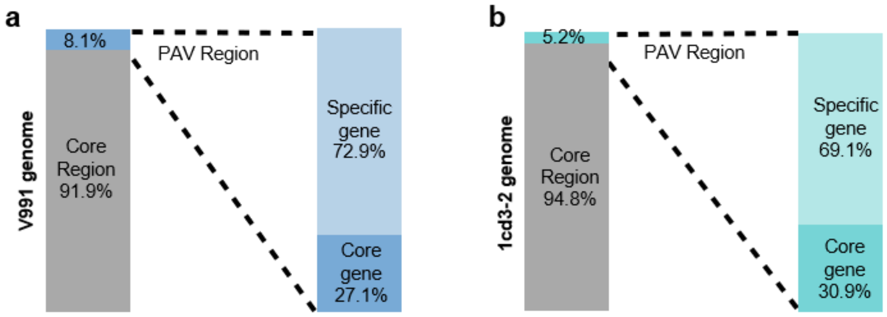

Supplementary Fig. 4. Small PAV regions contain a large number of specific genes through whole genomes. a The proportion of specific genes and core genes in the PAV region of the V991 genome. **b** The proportion of specific genes and core genes in the PAV region of the 1cd3-2 genome. Source data are provided as a Source Data file.

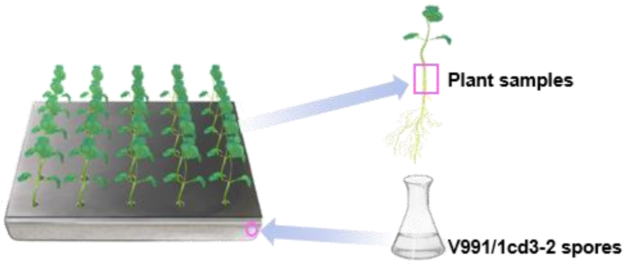

Supplementary Fig. 5. The sampling mode diagram of bidirectional transcriptome. The sampling mode diagram of bidirectional transcriptome on hypocotyls of cotton during the whole period of interaction (0, 3, 6, 9, 12, 15, 18, 21 days post-infection (dpi)). Total RNA was extracted from spore or infected cotton hypocotyls at 0,3,6,9,12,15,18 and dpi. The spores of the two pathogens cultured in liquid medium (Czapek-Dox) for 3 days and the hypocotyls without *V. dahliae* were used as the control. All samples were set with three independent biological replicates.

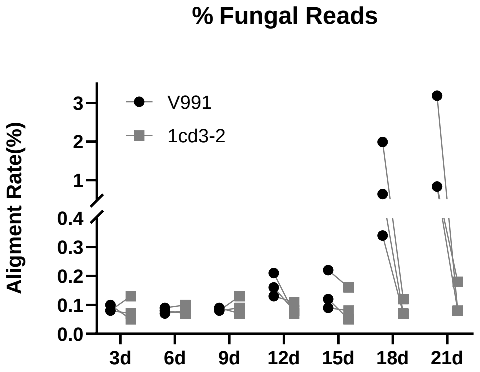

Supplementary Fig. 6. Ratio of V991 and 1cd3-2 reads identified at different time points in the transcriptomes. Source data are provided as a Source Data file.

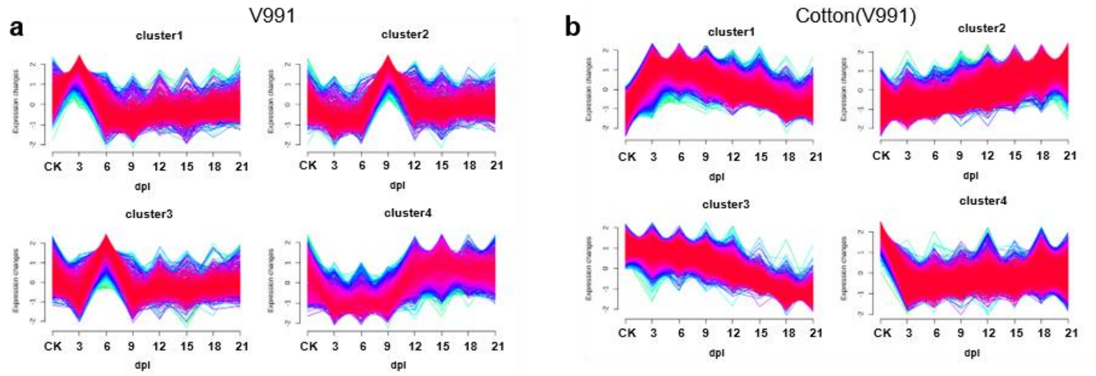

Supplementary Fig. 7. Identification of classes of differentially expressed genes in transcriptomes. a Applying the fuzzy c-means algorithm to cluster 2796 differentially expressed genes in V991 transcriptomes. Clusters 1, 2, and 3 represent genes that are upregulated in stage I (type1), clusters 4 represent genes that are upregulated in stage II (type2). **b** Applying the fuzzy c-means algorithm to cluster 14940 differentially expressed genes in cotton (V991) transcriptomes. Clusters 1 represent genes that are upregulated in stage I (type1), clusters 2 represent genes that are upregulated in stage II (type2). Fuzzy c-means clustering identified 4 distinct temporal patterns of RNA expression. The x axis represents 8 time points, while the y axis represents log2-transformed, normalized intensity ratios in each time point. Source data are provided as a Source Data file.

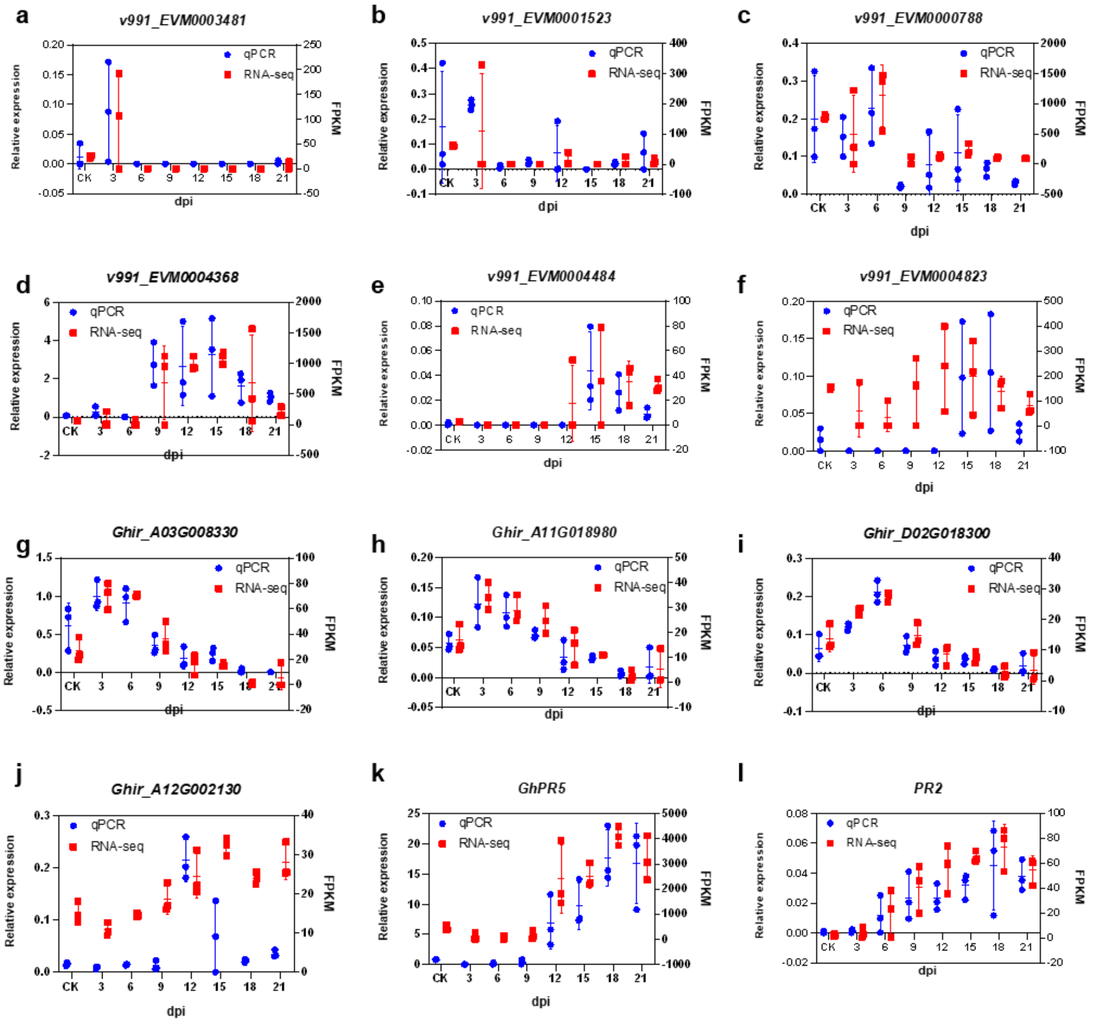

Supplementary Fig. 8. Validation of gene expression patterns by qRT-PCR. a-c The relative expression level of small molecule metabolic related genes in V991 transcriptome. **d-f** The relative expression level of developmental related genes in V991 transcriptome. *v991_EVM0005718* was used as an internal reference of *V.dahliae*. **g-i** The relative expression level of cell wall biogenesis related genes in cotton (V991) transcriptome; **j-l** The relative expression level of defense response related genes in cotton (V991) transcriptome. *GhUB7* was used as an internal reference of cotton. *x*-axis: Days Post Infection, the *y*-axes indicate relative expression values (left) and FPKM values (right). In **a-l**, the values are the means ± s.d. for three biological replicates. Each dot represents individual data points. Source data are provided as a Source Data file.

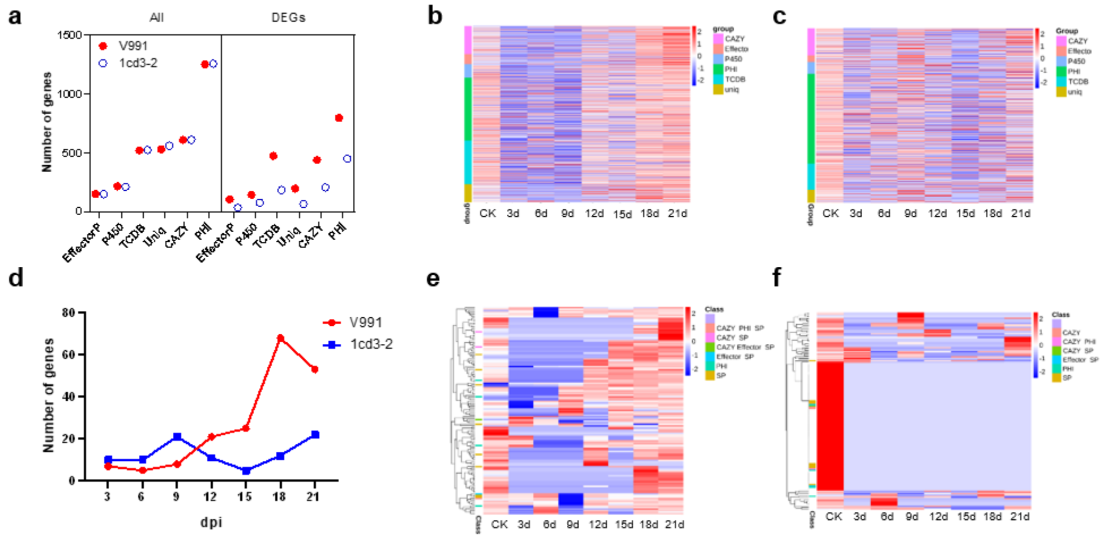

Supplementary Fig. 9. Gene number statistics and gene expression analysis in V991 and 1cd3-2 transcriptomes, respectively. a Statistics of genes of different categories at the genome level (left) and transcription level (right) in V991 and 1cd3-2, respectively. **b, c** Expression profiling of pathogenicity-related genes in V991 and 1cd3-2 transcriptomes, respectively. **d** The number of DEGs of V991 and 1cd3-2 in the PAV regions at different time points, respectively. **e, f** Expression profiling of genes in the PAV region in V991 and 1cd3-2 transcriptomes, respectively. Source data are provided as a Source Data file.

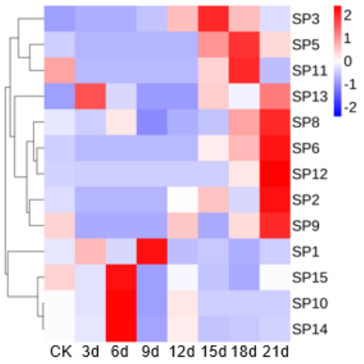

Supplementary Fig. 10. Expression profiling of secretory proteins in the PAV region in the V991 transcriptome. Source data are provided as a Source Data file.

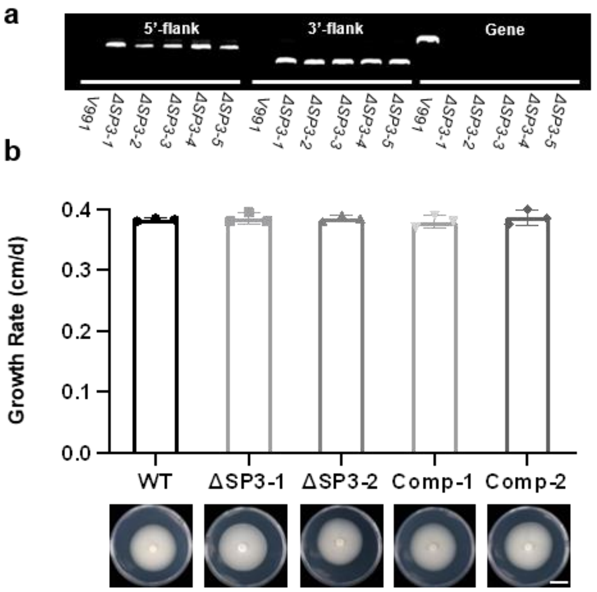

Supplementary Fig. 11. The growth rate of the wild-type V991, *ΔSP3* mutant, and *ΔSP3*/SP3-complementation transformants. a PCR analysis of wild type strain V991 and knockout mutants. The genomic DNA of each strain was used to verify the 5' and 3' homologous and presence of targeted gene. **b** The growth rate and radial growth of V991 (WT), knockout mutants and complemented transformants on PDA agar plates. Representative cultures at 5 days post-incubation were shown. The scale bar stands for 2 cm. The experiments were repeated three times with similar results. n = 3, n shows the number of samples. Each dot represents individual data points. No significant differences compared with the WT were found (Student's t test). Source data are provided as a Source Data file.

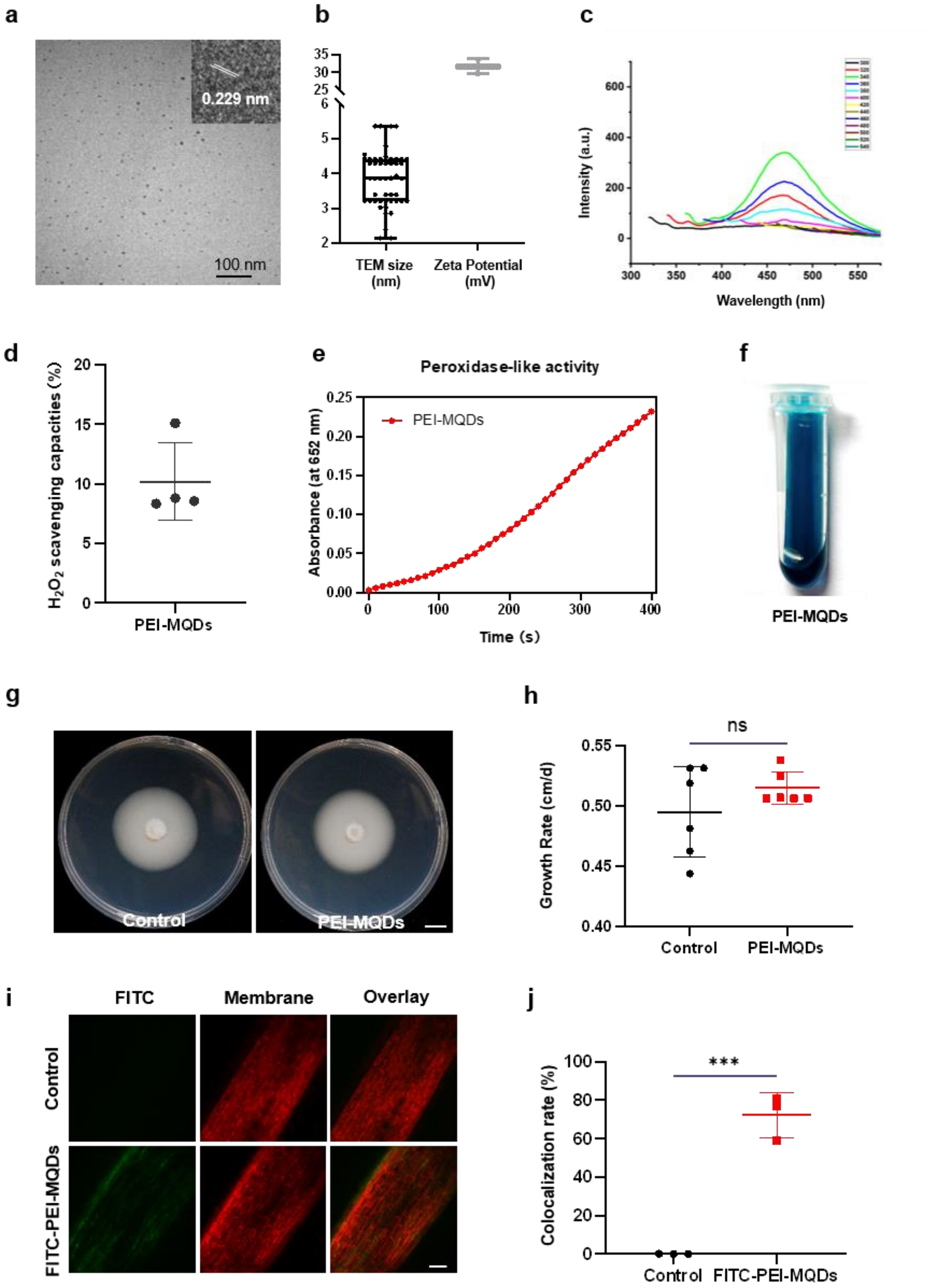

Supplementary Fig. 12. The characterization PEI-MQDs. a The TEM image of PEI-MQDs. **b** The zeta potentional and the TEM size of PEI-MQDs. **c** The emission wavelength of PEI-MQDs in different bands. **d-f** *In vitro* test of PEI-MQDs peroxidase-like catalytic activity. In each box and whisker plot, the center line is the median. The bottom and top edges of the boxes indicate the twenty-fifth and seventy-fifth percentiles. Each dot represents individual data points. **d** H_2O_2

scavenging ability of PEI-MQDs. PEI-MQDs were exposed with H_2O_2 solution for 24 h, then PEI-MQDs were isolated and used for the following H_2O_2 scavenging activity. The values are the means ± s.d. for four biological replicates. *n* = 4, *n* shows independent experiments. Each dot represents individual data points. **e** Reaction–time curves of TMB colorimetric reaction catalyzed by PEI-MQDs. **f** PEI-MQDs catalyze the oxidation of peroxidase substrates (TMB) to produce colorimetric reactions. **g-h** Identification of growth rate of V991 on PDA agar plates without (control) and with PEI-MQDs treatment, respectively. Representative cultures at 4 days post-incubation were shown. The scale bar stands for 1 cm. The values are the means ± s.d.. All of the experiments were repeated at least three times with similar results. Statistical analyses were performed using a Student's *t* test: ns, Not significant. Each dot represents individual data points. **i** Confocal imaging results showed colocalization of membrane autofluorescence with FITC-PEI-MQDs in the root of the cotton. PEI-MQDs were labelled with FITC. The scale bar stands for 20 μm. **j** The calculated colocalization rate between FITC-PEI-MQDs and membrane. The values are the means ± s.d. for three biological replicates. The asterisks indicate statistically significant differences between the two groups (***$P<0.001$, Student’s *t*-test). Each dot represents individual data points. Source data are provided as a Source Data file.

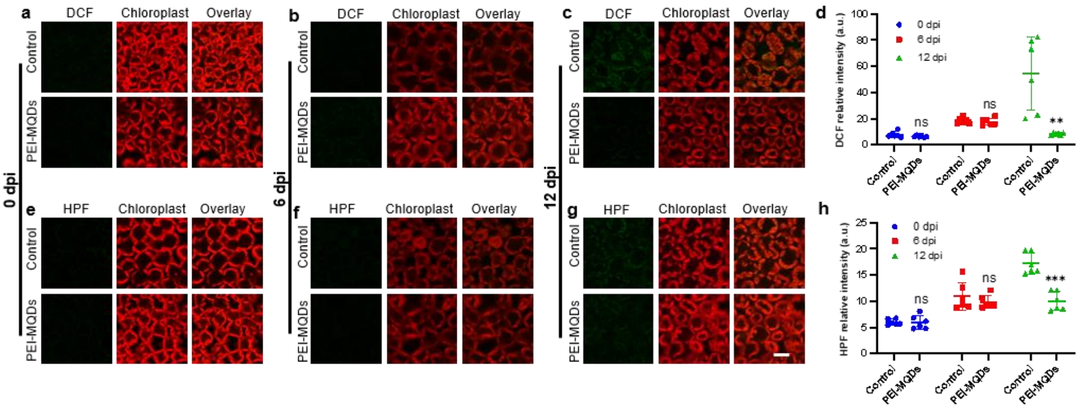

Supplementary Fig. 13. Confocal imaging of ROS fluorescent dyes in cotton at different time points. a-c The DCF (H_2O_2) fluorescent dye intensity in cotton leaves without (control) and with PEI-MQDs treatment at different time points, respectively. **d** The intensity calculation of DCF fluorescence. The values are means ± SD, *n* = 6. Statistical analyses were performed using a Student's *t* test: ns, Not significant; ***P*<0.01. **e-g** The HPF ($OH^{\bullet}$) fluorescent dye intensity in cotton leaves without (control) and with PEI-MQDs treatment at different time points, respectively. **h** The intensity calculation of HPF fluorescence. The scale bar stands for 50 μm. In **d** and **h**, the values are the means ± s.d.. All of the experiments were repeated at least three times with similar results. *n* = 6, *n* shows the number of leaf samples. Statistical analyses were performed using a Student's *t* test: ns, Not significant; ***P*<0.01; ****P*<0.001. Each dot represents individual data points. Source data are provided as a Source Data file.

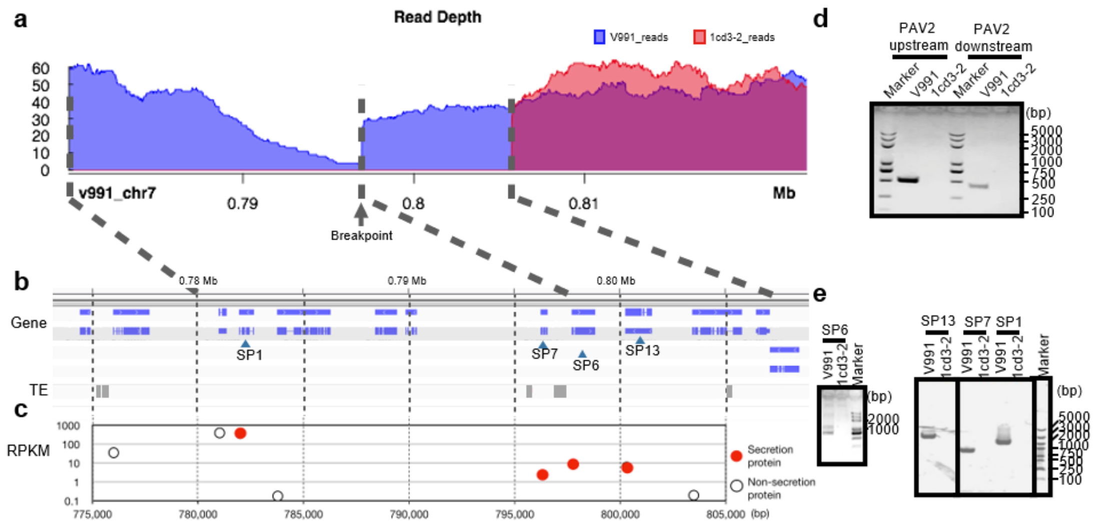

Supplementary Fig. 14. PAV incidents contribute to the formation of specific gene. a-e On chromosome 7, two adjacent insertion regions totally contain 10 genes including 8 specific genes, and 4 of them are secretion proteins. **a** PAV region was verified by PacBio sequence reads separately mapping to two genomes. **b** Gene and TE distribution in the PAV region. **c** Maximum gene regulation level for each gene from upper panel. PCR validation of PAV regions (**d**) and specific genes (**e**) (*SP1*, *SP6*, *SP7*, *SP13*). Source data are provided as a Source Data file.

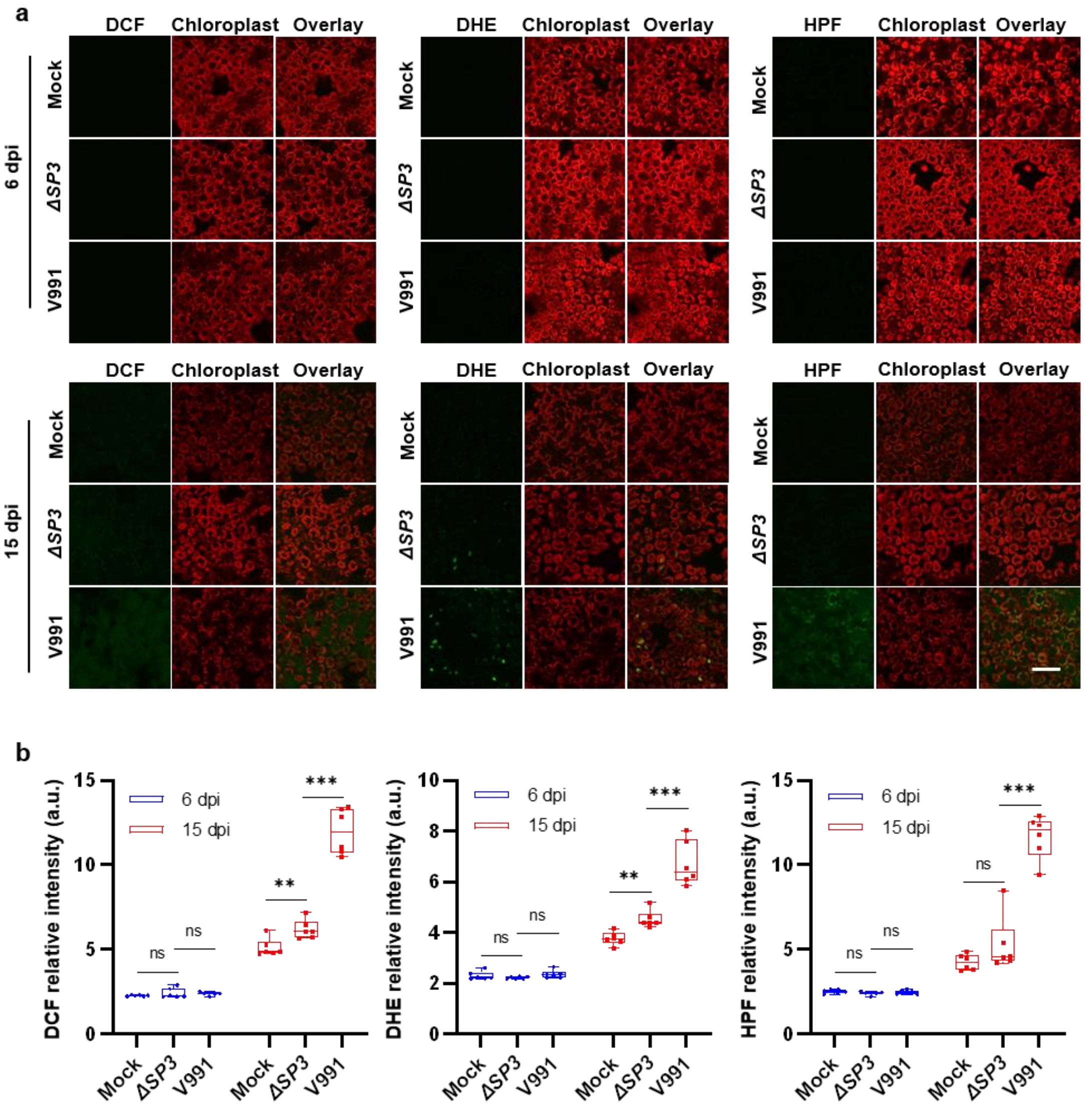

Supplementary Fig. 15. The ROS content decreased in leaves of cotton infected with SP3 mutant. a Confocal imaging of DCF (H_2O_2), DHE ($^{\bullet}O_2^-$) and HPF ($OH^{\bullet}$) in cotton leaves infested with H_2O (Mock), *ΔSP3* and V991 at different time points, respectively. The scale bar stands for 50 μm. **b** Quantitative analysis of fluorescence intensity of leaf DCF (H_2O_2), DHE ($^{\bullet}O_2^-$) and HPF ($OH^{\bullet}$) in cotton infested with H_2O (Mock), *ΔSP3* and V991 at different time points, respectively. The values are the means ± s.d. for six biological replicates. n = 6, n shows the number of leaf samples. The asterisks indicate statistically significant differences between the two groups (ns, Not significant; **P<0.01; ***P<0.001, Student's t-test). In each box and whisker plot, the center line is the median. The bottom and top edges of the boxes indicate the twenty-fifth and seventy-fifth percentiles. Each dot represents individual data points. Source data are provided as a Source Data file.

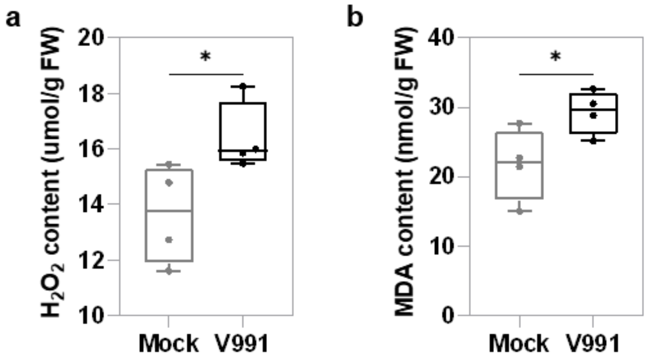

Supplementary Fig. 16. The H_2O_2 and MDA content in V991-infested cotton leaves. The H_2O_2 content (**a**) and the MDA content (**b**) were measured in cotton leaves inoculated with V991 for 12 days compared with those of uninoculated cotton leaves (Mock). **T**he values are the means ± s.d. for four biological replicates. The asterisks indicate statistically significant differences between the two groups (*P<0.05, Student's *t*-test). In each box and whisker plot, the center line is the median. The bottom and top edges of the boxes indicate the twenty-fifth and seventy-fifth percentiles. Each dot represents individual data points. Source data are provided as a Source Data file.

Supplementary Table 1. Summary of genome assembly and annotation for *V. dahliae*.

Genomic feature	V991	1cd3-2
Total scaffold (Mb)	35.7	34.5
Total contig (Mb)	35.7	34.5
Number of scaffolds	13	19
Number of contigs	13	19
Largest contig (Mb)	7.8	6.7
Scaffold N50* (Mb)	3.8	3.5
Contig N50* (Mb)	3.8	3.5
Total gap (Kb)	0	0
GC content (%)	53.8	53.8
Total size of TE (Mb)	3.4	2.6
Protein-coding genes	10941	10971
Mean gene length (bp)	1855	1852
rRNA genes	83	72
tRNA genes	267	271
ncRNAs	36	35

Supplementary Table 2. Summary of core and specific genes, secreted proteins (SPs), putative effectors.

Genome	V991	1cd3-2
Core genes	10411	10411
Core SPs	1068	1071
Core Effectors	108	110
Speciic genes	530	560
Specific SPs	26	32
Specific Effectors	8	7

Supplementary Table 3. The average frequency of specific genes distribution in different genomic region.

**Core/SP/whole**	**Genome**	**Core region**	**Whole genome**	**PAV region**
Specific gene number	V991	309	530	221
	1cd3-2	370	560	190
Genome size (Mb)	V991	32.8	35.7	2.9
	1cd3-2	32.7	34.5	1.8
Distribution Fre	V991	9.4	14.8	76.2
	1cd3-2	11.3	16.2	105.6

Supplementary Table 4. Predicted secreted proteins present in V991 but absent in 1cd3-2 by PAV.

Name	Gene ID	Protein length	No. of cysteines	NLS sequence	Annotation
SP1	v991_EVM0000309	144	2		Putative uncharacterized protein
SP2	v991_EVM0001062	278	5		Putative uncharacterized protein
SP3	v991_EVM0003170	372	1		Cupin domain Quercetin 2,3-dioxygenase
SP4	v991_EVM0003245	398	8		Predicted protein
SP5	v991_EVM0003848	705	11		NACHT domain Vegetative incompatibility protein HET-E-1
SP6	v991_EVM0005110	358	3	PPDPKRRIEVI	GDSL-like Lipase/Acylhydrolase family
SP7	v991_EVM0005218	85	2		Putative uncharacterized protein
SP8	v991_EVM0005425	207	0		Endo-1,4-beta-xylanase activity
SP9	v991_EVM0005614	1071	17		Protein of unknown function
SP10	v991_EVM0006631	107	2		Putative uncharacterized protein
SP11	v991_EVM0009178	474	11		Predicted protein
SP12	v991_EVM0009773	389	6		Pectate lyase
SP13	v991_EVM0010514	375	1		Lyase activity
SP14	v991_EVM0010728	107	2		Putative uncharacterized protein
SP15	v991_EVM0006135	163	3		Putative uncharacterized protein

Signal peptide was predicted by SignalP (http://www.cbs.dtu.dk/services/SignalP/).
Nuclear localization signal (NLS) sequence was predicted by cNLS Mapper (http://nls-mapper.iab.keio.ac.jp/cgi-bin/NLS_Mapper_form.cgi)

Supplementary Table 5. The ROS and RNS scavenging capacities of PEI-MQDs *in vitro.*

Material	Concentration	Clearance rate			
PEI-MQDs	50ppm	•O_2^-	H_2O_2	OH•	$ONOO^-$
		11.70%	6.56%	42.93%	27.23%
