# Supplementary material for: Polyethyleneimine-coated MXene quantum dots improve cotton tolerance to Verticillium dahliae by maintaining ROS homeostasis
Source: Nat Commun. 2023 Nov 15;14:7392. doi: 10.1038/s41467-023-43192-4 (PMC10651998; doi:10.1038/s41467-023-43192-4)
Supplement: Supplementary file 2 — Description of Additional Supplementary Files [file 41467_2023_43192_MOESM2_ESM.pdf]

File Name: Supplementary Data 1
Description: GO enrichment analysis of up-regulated DEGs at stage I in V991 transcriptome.

File Name: Supplementary Data 2
Description: GO enrichment analysis of up-regulated DEGs at stage II in V991 transcriptome.

File Name: Supplementary Data 3
Description: GO enrichment analysis of up-regulated DEGs at stage I in cotton (V991) transcriptome.

File Name: Supplementary Data 4
Description: GO enrichment analysis of up-regulated DEGs at stage II in cotton (V991) transcriptome.

File Name: Supplementary Data 5
Description: Primers used in the study.
