# Supplementary material for: Polyethyleneimine-coated MXene quantum dots improve cotton tolerance to Verticillium dahliae by maintaining ROS homeostasis
Source: Nat Commun. 2023 Nov 15;14:7392. doi: 10.1038/s41467-023-43192-4 (PMC10651998; doi:10.1038/s41467-023-43192-4)
Supplement: Supplementary file 8 — Reporting Summary [file 41467_2023_43192_MOESM8_ESM.pdf]

nature portfolio

Corresponding author(s): HONGHONG WU

Last updated by author(s): Oct 14, 2023

Reporting Summary

Nature Portfolio wishes to improve the reproducibility of the work that we publish. This form provides structure for consistency and transparency in reporting. For further information on Nature Portfolio policies, see our Editorial Policies and the Editorial Policy Checklist.

Statistics

For all statistical analyses, confirm that the following items are present in the figure legend, table legend, main text, or Methods section.

n/a	Confirmed	
☐	☒	The exact sample size (*n*) for each experimental group/condition, given as a discrete number and unit of measurement
☒	☐	A statement on whether measurements were taken from distinct samples or whether the same sample was measured repeatedly
☐	☒	The statistical test(s) used AND whether they are one- or two-sided<br>*Only common tests should be described solely by name; describe more complex techniques in the Methods section.*
☒	☐	A description of all covariates tested
☒	☐	A description of any assumptions or corrections, such as tests of normality and adjustment for multiple comparisons
☐	☒	A full description of the statistical parameters including central tendency (e.g. means) or other basic estimates (e.g. regression coefficient) AND variation (e.g. standard deviation) or associated estimates of uncertainty (e.g. confidence intervals)
☐	☒	For null hypothesis testing, the test statistic (e.g. *F*, *t*, *r*) with confidence intervals, effect sizes, degrees of freedom and *P* value noted<br>*Give P values as exact values whenever suitable.*
☒	☐	For Bayesian analysis, information on the choice of priors and Markov chain Monte Carlo settings
☒	☐	For hierarchical and complex designs, identification of the appropriate level for tests and full reporting of outcomes
☒	☐	Estimates of effect sizes (e.g. Cohen's *d*, Pearson's *r*), indicating how they were calculated

Our web collection on statistics for biologists contains articles on many of the points above.

Software and code

Policy information about availability of computer code

Data collection	Canu was used to assembly long reads for genome assembly; LTR_FINDER (version 1.05), MITE-Hunter (20100819), RepeatScout (version 1.0.5) and PILER-DF (version 2.4) were utilized to generate repeat libraries based on structure-based method and de novo prediction; PASTEClassifier (version 1.0) was applied for repeat classification, then merged with all repeats from the Repbase Database (version 19.06); RepeatMasker was used to predict fungal isolate repeats; EVM was used to integrate all predicted results; Augustus, GlimmerHMM, and SNAP (version 2006-07-28) to scan the repeat-masked genome; PASA were used to predict genes ; the 6mA and 4mC base modifications were identified using PacBio SMRT Analysis 2.3.0 with default settings; The program Nucmer in Mummer was used to compare two genomes.
Data analysis	No commercial, open source and custom code used to analyse the data in this study were used.

For manuscripts utilizing custom algorithms or software that are central to the research but not yet described in published literature, software must be made available to editors and reviewers. We strongly encourage code deposition in a community repository (e.g. GitHub). See the Nature Portfolio guidelines for submitting code & software for further information.

Data

Policy information about availability of data

All manuscripts must include a data availability statement. This statement should provide the following information, where applicable:

- Accession codes, unique identifiers, or web links for publicly available datasets
- A description of any restrictions on data availability
- For clinical datasets or third party data, please ensure that the statement adheres to our policy

All supporting data from this study are available in the article and supplementary information files, or from the corresponding author upon reasonable request. The raw V991 and 1cd3-2 genome sequencing in this study have been deposited in the NCBI Bioproject database under accession code PRJNA510201. The V991 and 1cd3-2 genome assembly data generated in this study have been deposited in the Genome Warehouse in National Genomics Data Center, Beijing Institute of Genomics, Chinese Academy of Sciences / China National Center for Bioinformation, under accession number PRJCA020793 and PRJCA020800. The raw RNA-seq data generated in this paper have been deposited in the Genome Sequence Archive (Genomics, Proteomics & Bioinformatics 2021) in the National Genomics Data Center (Nucleic Acids Res 2022), China National Center for Bioinformation/Beijing Institute of Genomics, Chinese Academy of Sciences (GSA: CRA008562), which are publicly accessible.

Research involving human participants, their data, or biological material

Policy information about studies with human participants or human data. See also policy information about sex, gender (identity/presentation), and sexual orientation and race, ethnicity and racism.

Reporting on sex and gender	N/A
Reporting on race, ethnicity, or other socially relevant groupings	N/A
Population characteristics	N/A
Recruitment	N/A
Ethics oversight	N/A

Note that full information on the approval of the study protocol must also be provided in the manuscript.

Field-specific reporting

Please select the one below that is the best fit for your research. If you are not sure, read the appropriate sections before making your selection.

☒ Life sciences ☐ Behavioural & social sciences ☐ Ecological, evolutionary & environmental sciences

For a reference copy of the document with all sections, see nature.com/documents/nr-reporting-summary-flat.pdf

Life sciences study design

All studies must disclose on these points even when the disclosure is negative.

Sample size	we sequenced a 20 kb library to high depth (> 100X) using PacBio RS II . We found the determined genome size was almost 1 Mb larger (35.7Mb vs. 34.8Mb) (Chen et al., 2017). Scaffold number sharply decreased ca. 13 fold (13 vs. 167), and contig L50 sharply increased by 88-95 fold (V991 3.8 Mb and 1cd3-2 3.5 Mb, vs VdLs.17 0.04Mb) . By optimal homologue comparison with chromosomes of VdLs.17 (carried out by Faino et al., 2015), we finally determined the sequence of 8 chromosomes of V991 and 1cd3-2, constructed from 13 and 19 contigs.
Data exclusions	No data was excluded from the analysis.
Replication	These samples were selected for DNA sequencing and do not require replication.
Randomization	These samples were selected for DNA sequencing and do not require Randomization.
Blinding	These samples were selected for DNA sequencing and do not require Blinding.

Reporting for specific materials, systems and methods

We require information from authors about some types of materials, experimental systems and methods used in many studies. Here, indicate whether each material, system or method listed is relevant to your study. If you are not sure if a list item applies to your research, read the appropriate section before selecting a response.

Materials & experimental systems

n/a	Involved in the study	
☒	☐	Antibodies
☒	☐	Eukaryotic cell lines
☒	☐	Palaeontology and archaeology
☒	☐	Animals and other organisms
☒	☐	Clinical data
☒	☐	Dual use research of concern
☐	☒	Plants

Methods

n/a	Involved in the study	
☒	☐	ChIP-seq
☒	☐	Flow cytometry
☒	☐	MRI-based neuroimaging

Dual use research of concern

Policy information about dual use research of concern

Hazards

Could the accidental, deliberate or reckless misuse of agents or technologies generated in the work, or the application of information presented in the manuscript, pose a threat to:

No	Yes	
☒	☐	Public health
☒	☐	National security
☒	☐	Crops and/or livestock
☒	☐	Ecosystems
☒	☐	Any other significant area

Experiments of concern

Does the work involve any of these experiments of concern:

No	Yes	
☒	☐	Demonstrate how to render a vaccine ineffective
☒	☐	Confer resistance to therapeutically useful antibiotics or antiviral agents
☒	☐	Enhance the virulence of a pathogen or render a nonpathogen virulent
☒	☐	Increase transmissibility of a pathogen
☒	☐	Alter the host range of a pathogen
☒	☐	Enable evasion of diagnostic/detection modalities
☒	☐	Enable the weaponization of a biological agent or toxin
☒	☐	Any other potentially harmful combination of experiments and agents
